# Role of Tau Acetylation in Alzheimer’s Disease and Chronic Traumatic Encephalopathy: The Way Forward for Successful Treatment

**Published:** 2017-12-07

**Authors:** Brandon Lucke-Wold, Kay Seidel, Rub Udo, Bennet Omalu, Mark Ornstein, Richard Nolan, Charles Rosen, Joel Ross

**Affiliations:** 1 Department of Neurosurgery, West Virginia University School of Medicine, Morgantown, WV; 2 Dr. Senckenberg Chronomedical Institute, J. W. Goethe University, Frankfurt am Main, Germany; 3 Department of Pathology, University of California Davis Medical Center, Davis, CA; 4 Cogwellin LLC 4 Industrial Way W, Eatontown NJ, USA

**Keywords:** Tau acetylation, Neurodegenerative diseases, Pathologic changes, Secondary injury cascades, Novel treatment target

## Abstract

Progressive neurodegenerative diseases plague millions of individuals both in the United States and across the world. The current pathology of progressive neurodegenerative tauopathies, such as Alzheimer’s disease (AD), Pick’s disease, frontotemporal dementia (FTD), and progressive supranuclear palsy, primarily revolves around phosphorylation and hyperphosphorylation of the tau protein. However, more recent evidence suggests acetylation of tau protein at lysine 280 may be a critical step in molecular pathology of these neurodegenerative diseases prior to the tau hyperphosphorylation. Secondary injury cascades such as oxidative stress, endoplasmic reticulum stress, and neuroinflammation contribute to lasting damage within the brain and can be induced by a number of different risk factors. These injury cascades funnel into a common pathway of early tau acetylation, which may serve as the catalyst for progressive degeneration. The post translational modification of tau can result in production of toxic oligomers, contributing to reduced solubility as well as aggregation and formation of neurofibrillary tangles, the hallmark of AD pathology. Chronic Traumatic Encephalopathy (CTE), caused by repetitive brain trauma is also associated with a hyperphosphorylation of tau. We postulated acetylation of tau at lysine 280 in CTE disease could be present prior to the hyperphosphorylation and tested this hypothesis in CTE pathologic specimens. We also tested for ac-tau 280 in early stage Alzheimer’s disease (Braak stage 1). Histopathological examination using the ac tau 280 antibody was performed in three Alzheimer’s cases and three CTE patients. Presence of ac-tau 280 was confirmed in all cases at early sites of disease manifestation. These findings suggest that tau acetylation may precede tau phosphorylation and could be the first “triggering” event leading to neuronal loss. To the best of our knowledge, this is the first study to identify acetylation of the tau protein in CTE. Prevention of tau acetylation could possibly serve as a novel target for stopping neurodegeneration before it fully begins. In this study, we highlight what is known about tau acetylation and neurodegeneration.

## Introduction

Tau hyperphosphorylation and progression have long dominated the underlying dogma behind disease progression in Alzheimer’s disease (AD) and Chronic Traumatic Encephalopathy (CTE). Multiple treatment strategies have been employed to prevent tau hyperphosphorylation or tau deposition as well as preventing amyloid deposition in AD. Unfortunately, these flawed hypotheses have failed to produce meaningful treatment options that can benefit patients. Our group and others have shown that looking earlier in the disease process may be more valuable in finding a treatment solution that can be clinically successful. Secondary injury mechanisms such as oxidative stress, endoplasmic reticulum (ER) stress, and neuroinflammation play a more important role in disease onset than previously assumed [1–4]. These secondary injury mechanisms can be primed by genetic predisposition and triggered by insults such as neurotrauma, drug abuse, and cardiovascular disease [5–7]. Once activated, these cascades lead to persistent damage to neurons and surrounding glia causing distinct tau acetylation [8]. It is at this crucial stage that the pivot point occurs on whether the brain recovers or progresses to neurodegeneration. Tau acetylation has been shown to both disengage tau from the microtubule and also facilitate tau aggregation [9]. Because of this, preventing tau acetylation is critical for stopping disease onset. Salsalate and methylene blue have both been shown to reduce tau acetylation in pre-clinical models, however the exact mechanism has not been fully elucidated and warrants further investigation [10]. It is likely that these drugs are limiting the expansion of secondary injury cascades following insult. In this review, we highlight the relationship of tau acetylation to AD and CTE and then discuss the most effective strategy for reducing tau acetylation via pharmaceutical intervention.

### Current Understanding of AD

On November 3, 1906, Alois Alzheimer presented the first definitive microscopic evidence of the tau tangle pathology that has become characteristic of degenerative AD [11]. By using the same stain that Max Bielschowsky used four years earlier, he also described neuritic plaques that sparked the amyloid hypothesis years later. The pathology was accompanied by detailed clinical reports of progressive dementia for several years prior to death. These findings would set the framework of defining AD. In 1984 the amyloid protein was identified as the core of the neuritic plaque and the amyloid cascade hypothesis was born. Over the past 33 years scientists have been attempting to find safe and effective treatments to remove amyloid from the brains of AD patients. Pharmaceutical companies have been testing agents to slow production of amyloid as well as administering antibodies or vaccines to remove amyloid from the brains of AD patients. Sadly, every anti-amyloid study to date has failed [12].

Proponents of the amyloid hypothesis still hold out hope that if such agents are administered at the asymptomatic stage or at the very early mild cognitive impairment stage positive results with acceptable side effects might be achieved [13]. More recently there has been a shift to look at pathologic tau in the progression of AD. It is the location and amount of tau found on autopsy in AD subjects that correlates best with stage and severity of symptomatology, not the amyloid plaque [14]. As far back as 1963, such tangles were noted to be composed of filaments with a diameter of approximately 10 nm that have come to be known as paired helical filaments (PHF) [15]. In 1992, Dreschel et al. first reported on the structural importance of the micro-tubular associated protein, tau in disease pathology [16].

It was at this point that research shifted towards understanding tau post-translational modifications. Normally tau has 83 potential sites of phosphorylation at various serine, threonine, and tyrosine sites of the 441 amino acid long tau protein. Excess p-tau is as high as 4–5× the normal level in the brain homogenates of AD brains when compared to control brains [17]. Because tau hyperphosphorylation was the first characterized, it garnered the most attention. In 1994, Khalid Iqbal was first to report that tau is a marker of neurofibrillatory tangles in AD patients upon autopsy [18]. He and others noted that an excess phosphorylation (p-tau) of the human tau protein could be pathological [17]. In subsequent studies of AD and related “abnormal” tau associated disorders such as corticobasilar degeneration, progressive supranuclear palsy, Picks disease and chronic traumatic encephalopathy, excess of tau phosphorylation have been consistently reported [19]. Recently however evidence has emerged that p-tau is not pathogenic and is not responsible for the loss of neuronal function in AD [20].

### Current Understanding of CTE

CTE is a devastating neurodegenerative disease triggered by head injury. Like other tauopathies, it is progressive in nature and contributes to both cognitive and functional decline. Omalu et al. described the modern version of CTE in retired professional athletes (21), which have been validated by McKee et al. [22]. The players had extensive histories of repetitive concussions followed by a series of impulsive events, cognitive decline, and frequently suicide. This original characterization of CTE sparked huge controversy and met resistance by sports organizations and scientists alike. Over the next decade, other groups validated the findings proposed by Omalu and began describing a very distinct pathologic paradigm [23]. Similar to Alzheimer’s disease, the disease correlation was most in line with the tau progression but not the amyloid hypothesis.

Our group and others began looking towards secondary injury mechanisms that can contribute to tauopathy. Promising pre-clinical data pointed to the role of endoplasmic reticulum stress and oxidative stress in the pathophysiology of neurodegeneration following neurotrauma [2,24]. The exact mechanism by which this occurs however was not completely elucidated. We found that ER stress is increased in cells that are undergoing apoptosis as well as those that develop tauopathy [25]. The key markers that were increased were markers of tau hyperphosphorylation AT100, PHF, and CP-13 [26]. It is apparent that tau hyperphosphorylation is an end-stage marker. How these markers got increased and what maintained the progressive tauopathy is still under investigation. Kondo and colleagues proposed that cis tau might play a role [27]. Kayed et al. assert that it is more likely the tau oligomers [28]. We however have discovered an earlier contributor to the disease process in tau acetylation.

### The Tau Pathology Catalyst

The lack of correlation between tau phosphorylation and functional decline sparked interest in understanding which tau modification actually does contribute to pathology. Tau is a very soluble hydrophilic protein [29]. Full-length tau remains soluble in solution up to 10M before it can aggregate. The tau repeat domain needs a concentration in solution of 4M to aggregate. High p-tau expressed in Sf9 cells (high phosphate) requires 0.2M before p-tau can aggregate. Since the concentration of p-tau in the CSF 20 attomolar, 1 picomolar in the interstitial fluid, and 1.0 micromolar in the neurons, it would seem quite impossible to expect p-tau at the levels seen in human AD brains to aggregate without a catalyst [30]. Thus, there must be a nucleating factor that acts as the “seed” which leads to the phosphorylation seen in the neurofibrillatory tangle. A neurofibrillary tangle is composed of a “fuzzy coat” or “soft polymer brush” [31]. This coat surrounds the core of tau fibers and can bind multiple cell components. Inside the tangle is a rigid fibril core. There are spokes emanating out of the core where two post translationally modified protein motifs exist known as hexapeptide PHF6* and PHF6. PHF6* has the following amino acid sequence: V Q I N N K whereas PHF6 has the following amino acid sequence: V Q I V Y K. Both hexapeptides are in the microtubular binding domain (MTBD) portion of the tau protein and are thought to be essential for the proper binding of tau onto the alpha and beta tubulin subunits of the microtubule [32].

Recent evidence suggests that a posttranslational acetylated modification of lysine at position 280 of the hexapeptide of the PHF6 can lead to pathological aggregation of tau [33]. Post mortem mass spectrometry analysis of AD brains has shown such acetylation occurs most specifically at lysine position 280. However, acetylation of lysine at positions 174, 274 and 281 has also been reported in other human tauopathies [34]. This acetylation may be due to the overactivity of the acetyltransferase enzyme p-300, which acts specifically on PHF6. Gorsky et al showed that even pseudo-acetylation of the single K280 residue by p-300 was able to exacerbate hTau neurotoxicity in vivo, which is suggestive that acetylated tau contributes to the pathology seen in neurodegenerative diseases [35].

**Figure 1:**
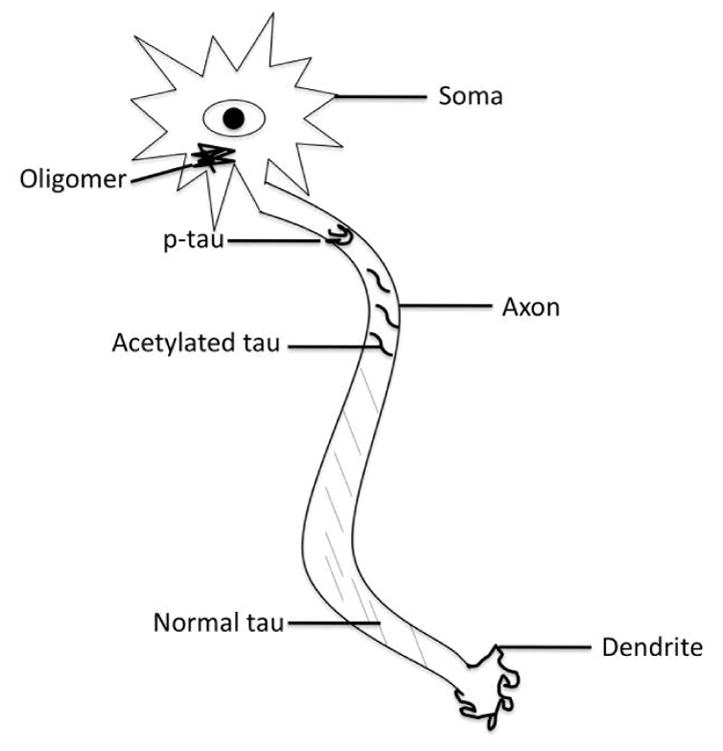
Schematic showing tau pathology progression up the axonal tracts. Tau becomes acetylated thereby exposing more phosphorylation sites. Once hyperphosphorylated tau aggregates into paired helical filaments, which ultimately produce tau oligomers and neurofibrillary tangles.

After tau is acetylated, there is dislodgement of the microtubular binding domain from the tubulin due to the neutralization of charges between tau and tubulin molecules. This exposes the previously unphosphorylated serines/threonines/tyrosines to kinases leading to the robust phosphorylation so often seen in brain homogenates of AD and CTE patients [36]. It is likely that the hyperphosphorylation will only occur in the context of tau acetylation. A shift in favor of the tau kinases over the phosphatases most likely occurs [37]. In Irwin’s seminal paper, there is co-localization of acetylated K280 with multiple p-tau epitopes in post-mortem AD brains [38]. Their study showed that acetylated K280 occurred early in the pathogenesis of neurodegeneration. Originally Braak and Braak indicated that phosphorylated tau in AD patients starts in the enterorhinal and transenterorhinal cortex and spreads up through the neocortex [39]. In 2012 Senanarong et al. reported a very early occurrence of AD-related cytoskeletal changes of p-tau in brainstem nuclei with likely spreading in a prion like manner (prionoid) up the white matter tracks to the neo/allocortex [40] (Figure 1). We highlight below that tau acetylation occurs in these exact same regions.

## Methods

Human paraffin embedded specimens were collected from post-mortem samples of the entorhinal cortex for CTE brains (N = 3) and from the putamen, caudate, thalamus, brain stem, and cerebral white matter of AD brains (N = 3). Control samples were selected from age and gender matched controls that succumbed to non-neurologic diseases. Brain slices were cut to 10 μm thickness with a Leica RM2265 microtome (Leica Biosystems). The slides were soaked in 99% formic acid for 10 minutes. A tau acetylation antibody for K280 was utilized. (Anaspec, rabbit polyclonal antibody). Staining of AD brain slices was conducted by Udo Rub and Kay Seidel at their laboratory of Goethe-University, Frankfurt/Main, Germany. The antibody was used at a concentration of 1:200. The standardized method of staining with primary and secondary antibodies was performed as has previously been published [2]. CTE brains were co-stained with MC1, which was kindly gifted from Dr. Peter Davies. The CTE images were analyzed with the Just another Co-localization plug-in from Image J. An overlap coefficient was generated for each overlay. R > 0.8 indicates strong correlation, R = 0.6–0.8 equals moderate correlation, R = 0.4–0.6 = weak correlation, and R < 0.4 is minimal overlay.

## Results

### AD Brains

**Figure 2:**
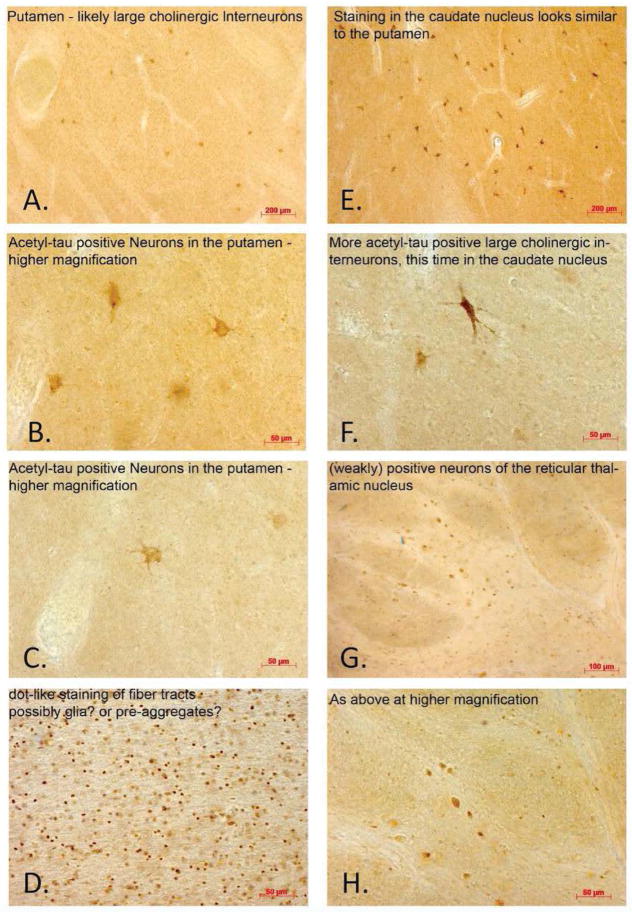
Tau acetylation at K280 in the putamen (A–D), caudate (E–F), and thalamus (G–H). These regions were chosen due to their known association with AD progression.

**Figure 3:**
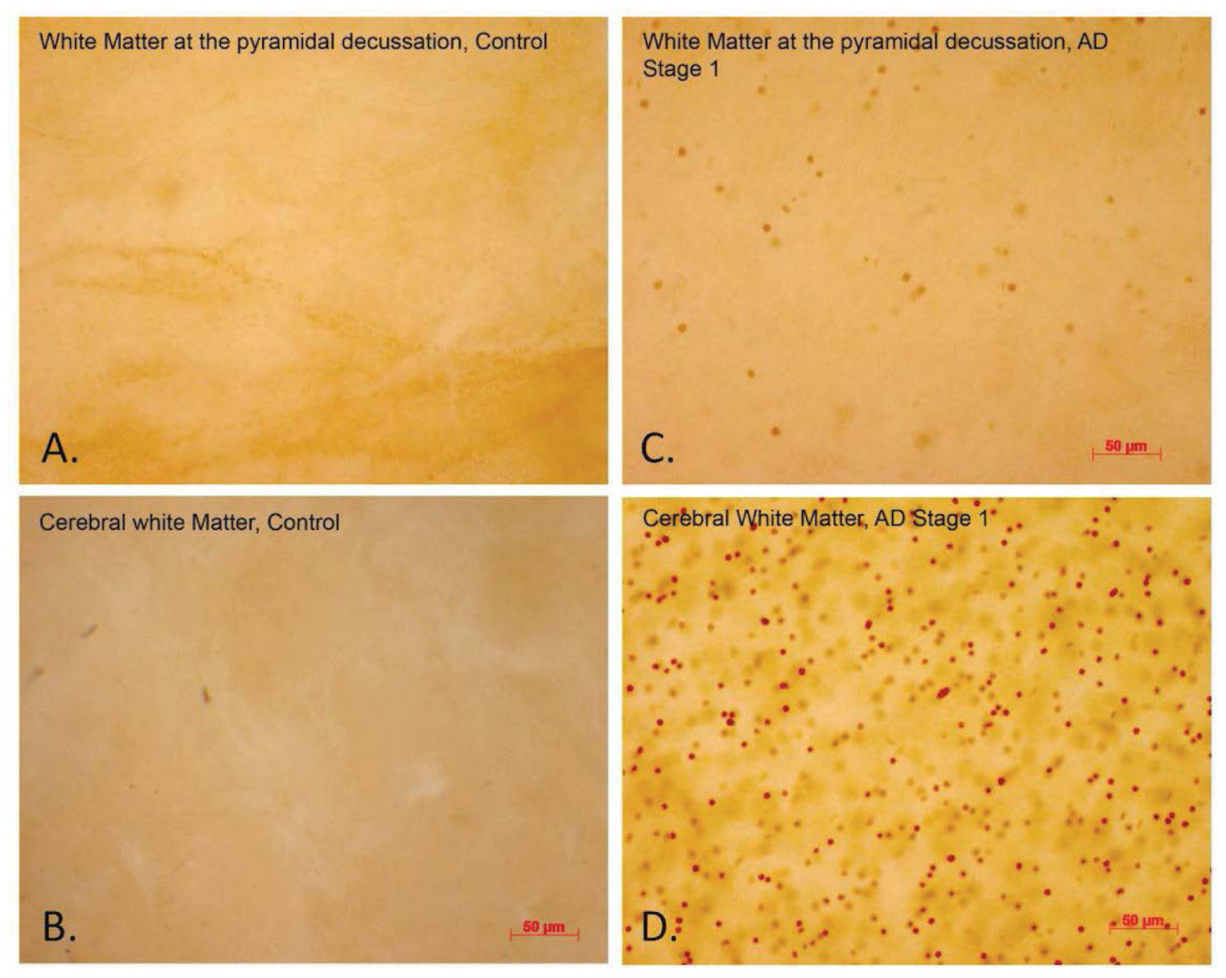
Tau acetylation at K280 in the brainstem of a control brain (A–B) and AD Braak Stage 1 (C–D). Tau acetylation was significantly more elevated in the AD brain verse the control.

Three Braak stage 1 brains were stained with the tau K280 acetylation antibody as well as matched controls. Significant staining was seen in the brain stem, caudate, and putamen (Figure 2). These regions were specifically chosen because of their importance for disease progression [41]. Slightly less staining was seen in the thalamus. Such early staining likely indicates the initial pathology in disease progression, which proceeds tau hyperphosphorylation. Similarly, staining was positive in the brainstem but was not seen in controls (Figure 3). Therefore, a strategy to slow tau acetylation seems plausible as a method to prevent disease onset and progression.

### CTE Brains

**Figure 4:**
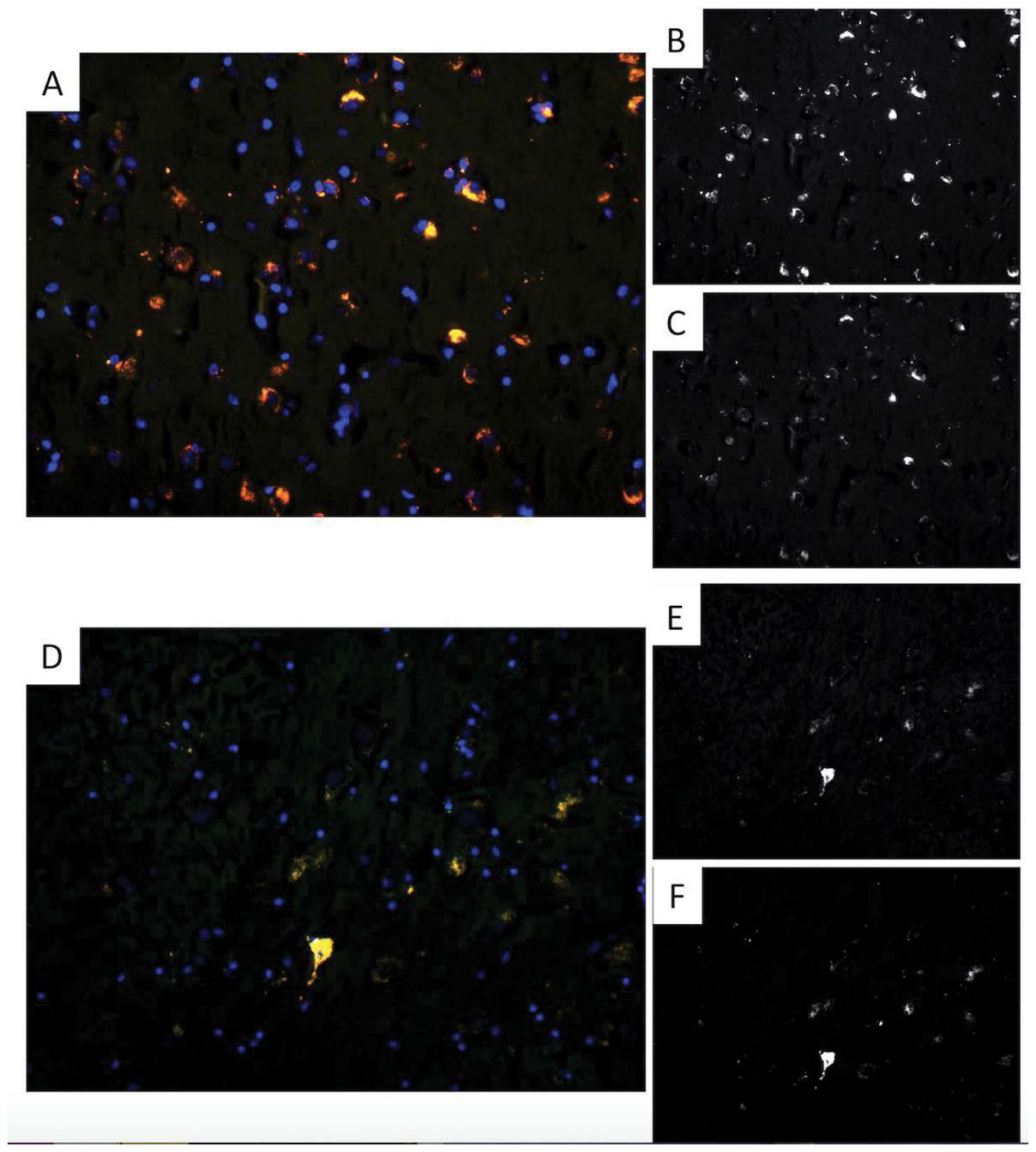
overlay of tau acetylation at K280 and phosphorylation (MC1) in entorhinal cortex of control brains. A) Overlay image at 20×, B) Acetylated tau K280 at 20×, C) MC1 at 20×. Overlay of tau acetylation at K280 and phosphorylation (MC1) in entorhinal cortex of CTE brains. D) Overlay image at 20×, E) Acetylated tau K280 at 20×, F) MC1 at 20×. Acetylation occurs at the same time or even prior to early tau phosphorylation.

**Figure 5:**
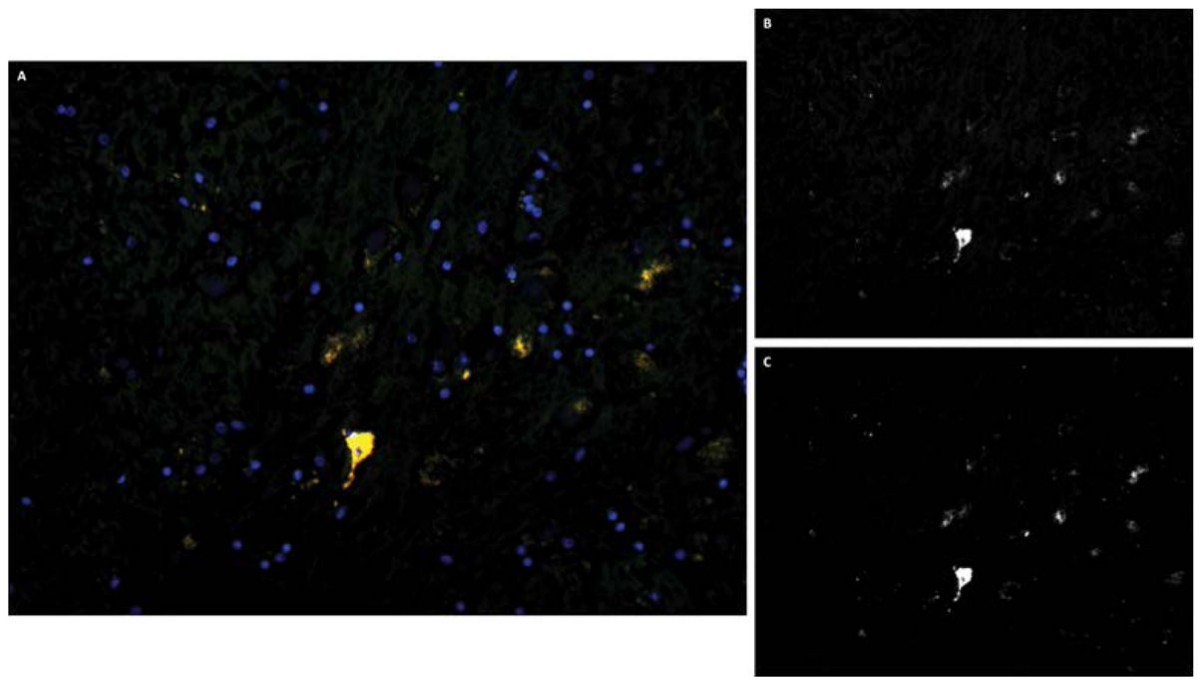
Overlay of tau acetylation at K280 and phosphorylation (MC1) in entorhinal cortex of CTE brains. A) overlay image at 20×, B) acetylated tau K280 at 20×, C) MC1 at 20×.

Three CTE brains were stained and compared to age-matched controls. An overlay between acetylated tau K280 and MC1 was done. MC1 is an early marker of tau hyperphosphorylation seen in neurodegeneration. Figure 4 shows an overlap coefficient of R = 0.33, which indicates minimal co-staining for tau pathology in control brains. Figure 5 shows an overlap coefficient of R = 0.97, which indicates strong correlation for tau acetylation and hyperphosphorylation in the entorhinal cortex. The results show that tau acetylation is at least present at time of tau phosphorylation, but more likely precedes it. This is important in that tau acetylation may serve as a potential pharmacologic target.

## Conclusions

Targeting tau phosphorylation has yielded little in terms of viable treatments for patients with neurodegeneration therefore urging a new strategy. Min and Gan from the Gladstone Institute tested the non-steroidal anti-inflammatory agent salsalate in pseudo-acetylated mice at a dose of 2.25 grams per day. The results indicated significant improvement in cognitive function due to reduction in p-300 induced tau acetylation and reduced hippocampal atrophy [42]. Lagraoui et al. further tested salsalate in a traumatic brain injury mouse model. Their report revealed that when salsalate was given post traumatic brain injury (TBI) there was a significant reduction in neuroinflammation, improved functional ability, as well as an upregulation of genes that are associated with neuroprotection and neurogenesis [43]. The likely mechanism is salsalate reducing ER stress and thereby limiting p-300 activity.

Going forward it is imperative to examine what actually drives disease pathogenesis. In this paper, we have outlined how tau acetylation plays a critical role in the process of neurodegeneration. Furthermore, we have shown that tau acetylation was increased both in human AD and CTE brain specimens. Future studies are warranted for how to target tau acetylation in pre-clinical models of AD and CTE in order to prevent disease progression and advance towards clinical trials. It is likely that ER stress, oxidative stress, and neuroinflammation are the triggers that activate the tau acetylation process. Pre-clinical work should flush out the mechanisms at play in order to advance towards clinical trials for potential treatments. An interdisciplinary team of clinicians and scientists will be necessary to tackle this important work and target the injury mechanisms as early as clinically detectable. Re-examining the tau hypothesis might be the paradigm shift needed for making headway on discovering new treatments for neurodegeneration.
